# Germacrone Inhibits Cell Proliferation and Induces Apoptosis in Human Esophageal Squamous Cell Carcinoma Cells

**DOI:** 10.1155/2020/7643248

**Published:** 2020-01-30

**Authors:** Ren Zhang, Ji Hao, Kaiwen Guo, Wanxin Liu, Fei Yao, Qingming Wu, Chang Liu, Qiang Wang, Xinzhou Yang

**Affiliations:** ^1^Institute of Infection, Immunology and Tumor Microenvironment, Hubei Province Key Laboratory of Occupational Hazard Identification and Control, Medical College, Wuhan University of Science and Technology, Wuhan 430065, China; ^2^School of Pharmaceutical Sciences, South-Central University for Nationalities, Wuhan 430074, China

## Abstract

Germacrone, a natural 10-membered monocyclic sesquiterpene with three double bonds and a ketone, was isolated from the roots of traditional Chinese medicine *Saussurea costus* (SC). The pharmacological value and intrinsic mechanism of germacrone in the treatment of esophageal squamous cell carcinoma (ESCC) are still unclear. Therefore, in this study, we further explored the internal molecular mechanism by which germacrone exerts its antiproliferation and antimigration ability against ESCC. 3-(4,5-Dimethylthiazol-2-yl)-2,5 diphenyltetrazolium bromide (MTT) assays showed that germacrone dose-dependently inhibited the proliferation of ESCC cells. Flow cytometry analysis (FACS) and wound healing experiments on germacrone treated ESCC cells showed that germacrone could induce apoptosis and inhibit the migration of ESCC cells in a dose-dependent manner. In the study on the mechanism of action of germacrone in antiesophageal cancer, we found that germacrone increased the ratio of Bax/Bcl-2 in the cytoplasm of ESCC, resulting in the activation of Caspase-9 and Caspase-3 and decreased the expression of Grp78, thereby reducing the inhibition of Caspase-12 and Caspase-7. In addition, we found that germacrone also inhibited STAT3 phosphorylation in a dose-dependent manner. In conclusion, we determined that germacrone exerted an antiesophageal effect through intrinsic apoptotic signaling pathways and by inhibiting STAT3 activity in ESCC cells.

## 1. Introduction

Esophageal cancer is the ninth most common cancer in the world. Types of esophageal cancer include esophageal squamous cell carcinoma (ESCC) and esophageal adenocarcinoma (EAC) [1]. About 572,000 new cases of esophageal cancer are diagnosed each year and over 509,000 deaths are estimated to be due to esophageal cancer [1]. Its incidence was significantly affected by regional and ethnic differences [2]. The 5-year survival rate of patients with ESCC was only 10% [3]. In 2012, the number of deaths due to ESCC accounted for 5% of all cancer deaths [4]. Moreover, ESCC accounts for 80% of esophageal cancer cases worldwide and is the primary histological subtype [5]. At present, there are no effective chemopreventive and therapeutic strategies for this lethal disease. Since there are no early symptoms, ESCC is commonly diagnosed at an advanced stage. Moreover, poor efficacy, adverse drug reactions, and drug resistance are the biggest drawbacks to systemic chemotherapy of ESCC. Therefore, clarification of its pathogenesis and identification of efficacious agents as new potential chemotherapeutic remedies for its prevention, diagnosis, and treatment are urgently needed.

Plant-derived natural products provide a major source of anticancer agents with high efficiency and low toxicity. Many antitumor drugs are obtained directly or indirectly from natural products, such as camptothecin, paclitaxel, and doxorubicin, all of which have been successfully used in clinical practice [6]. In addition, a large number of anticancer agents from natural products are undergoing preclinical evaluation and clinical studies [7]. Hence, exploring more natural products from natural sources to treat ESCC may meet the growing demand for development of chemotherapy agents.

*Saussurea costus* (Falc.) Lipech (SC), a well-known traditional Chinese medicine, has long been used to treat asthma, certain bronchitis, ulcer, and stomach problems [8, 9]. Several reports indicated that the plant has hepatoprotective, antiparasitic, antiulcer, immunomodulatory, and anticancer properties [10]. In recent years, it has drawn wide attention due to its potential anticancer activities against various types of cancers. The main chemical components of SC are monoterpenoids and sesquiterpenoids [11]. Germacrone, a natural 10-membered monocyclic sesquiterpene with three double bonds and a ketone, is one of the main chemical constituents of the roots of SC. Germacrone can inhibit the proliferation of many cancers, such as glioma [12], retinoblastoma [13], breast cancer [14–16], liver cancer [17], prostate cancer [16], and colon cancer [16]. However, few researches about the effect of germacrone on ESCC cells have been reported so far. Hence, the object of the present study is to investigate the potential value of germacrone in ESCC treatment. In this study, germacrone was purified from the roots of SC. The antiproliferation assay of germacrone on ESCC cells showed that germacrone time- and dose-dependently inhibited the proliferation of ESCC cells. Wound healing and FACS assays revealed that germacrone inhibited ESCC migration and induced ESCC apoptosis. Our further data indicated that the molecular mechanism for germacrone induced ESCC cell apoptosis was associated with the inhibition of STAT3 phosphorylation, as well as the activation of the intrinsic apoptosis signaling pathway.

## 2. Materials and Methods

### 2.1. Instruments

Semipreparative high performance liquid chromatography (HPLC) was performed on a Waters 2535 HPLC coupled with a 2998 photodiode array (PDA) detector and a 2707 automatic sampler (Waters, Milford, MA, USA). Separations were carried out on Thermo C18 columns (5 *μ*m, 10 × 250 mm; 5 *μ*m, 20 × 150 mm). High performance liquid chromatography-photodiode array detector-electrospray ionization mass spectrometry (HPLC-PDA-ESIMS) analysis was recorded on a Waters ACQUITY SQD MS system connected to a Waters 1525 HPLC fitted with a 2998 PDA detector. EIMS data were obtained with a MAT-95 mass spectrometer. NMR spectra were recorded on an AVANCE III 600 MHz spectrometer (Bruker BioSpin, Ettlingen, Germany). The HPLC-grade acetonitrile for chromatography was purchased from Tedia Corporation (Fairfield, OH, USA). Sephadex LH-20 gel was obtained from GE Health Care (Uppsala, Sweden).

### 2.2. Reagents and Chemicals

Dulbecco's modified Eagle's medium (DMEM), fetal bovine serum (FBS), phosphate-buffered saline (PBS), and antibiotics (1% penicillin/streptomycin) were obtained from Hyclone (Logan, UT, USA). Annexin V-FITC kit and PI kit were purchased from BD Pharmingen (San Diego, USA). 3-(4,5-Dimethylthiazol-2-yl)-2,5 diphenyltetrazolium bromide (MTT) was obtained from Sigma (Darmstadt, Germany). Caspase-3(9962), Caspase-9(9508), MMP-9(13667), Bax (5023), Bcl-2 (2870), PARP (9542), STAT3(12640), p-STAT3(4113), and *β*-actin(3700) were purchased from Cell Signaling Technology (Boston, USA). Caspase-7 (GTX102337), Caspase-12 (GTX132298), and Grp-78 (GTX113340) were purchased from GeneTex (Texas, USA).

### 2.3. Plant Material

The roots of *Saussurea costus* (Falc.) Lipech. (family Compositae) were collected from Wufeng County, Hubei province, China, in July 2015, and identified by Professor Dingrong Wan at School of Pharmaceutical Sciences, South-Central University for Nationalities (SCUN), Wuhan, China. A voucher specimen (No. SC0187) was deposited in the School of Pharmaceutical Sciences, SCUN, Wuhan, China.

### 2.4. Extraction and Isolation

Air-dried roots of SC (400 g) were ground into powders and then extracted by maceration with 80% ethanol (4 × 5 L, 3 days each) at room temperature. The solvent was evaporated under reduced pressure to give a residue (100 g). The residue was dispersed in water and extracted repeatedly with petroleum ether (4 × 1.0 L) to obtain a terpene-enriched fraction (15 g). Fourteen grams of this fraction was subjected to D101 macroporous resin column chromatography (CC) (300 g, Sinopharm Chemical Reagent Co., Ltd., Shanghai, China) and eluted sequentially with 20%, 40%, 60%, 80%, and 95% ethanol in water to yield five fractions. The 80% ethanol fraction (2.4 g) had the strongest inhibitory effect on ESCC cell proliferation. Part of the 80% ethanol fraction (2.3 g) was separated by Sephadex LH-20 CC using CH_2_Cl_2_-MeOH (1 : 2, v/v) containing 0.1% formic acid, followed by semipreparative HPLC (acetonitrile : water : formic acid, 0–20 min, 60 : 40 : 0.1 to 100 : 0 : 0.1; 20–25 min, 100 : 0 : 0.1) to afford germacrone (150 mg). The structure of this compound was determined by comparing its ^1^H-NMR and ^13^C-NMR data (Figures [Supplementary-material supplementary-material-1] and [Supplementary-material supplementary-material-1] in Supplementary Materials) with those reported in the literature [18].

### 2.5. Cell Culture

The following human cell lines are used in this study: the ESCC cell lines Eca109 and EC9706 and the normal esophageal epithelial cell line Het-1A. Eca109 cells were obtained from the Shanghai Institute of Biochemistry and Cell Biology (Shanghai, China). EC9706 and Het-1A cells were obtained from the State Key Laboratory of Molecular Oncology (Beijing, China). All cell lines were cultured in DMEM supplemented with 10% (v/v) heat-inactivated FBS and 1% penicillin/streptomycin at 37°C in a humidified atmosphere of 5% CO_2_.

### 2.6. Cell Viability Analysis

The viability of Eca109, EC9706, and Het-1A cells was analyzed by MTT assay [19, 20]. Cells were seeded in 96-well plates at a density of 1 × 10^5^ cells/well. After 24 h cultivation, cells were treated with various concentrations of germacrone (0, 7.5, 15, 22.5, 30, 37.5, and 45 *μ*g/mL) for 12 h, 24 h, and 48 h, respectively. Then 100 *μ*L of MTT (5 mg/mL) was added into each well. Following further incubation for 4 h, the formazan crystals were dissolved in DMSO (100 *μ*L/well) and the absorbance was measured at 562 nm with a Microplate Reader (Bio-Rad, CA, USA). The growth inhibition rate was calculated with the following equation: Inhibition (%) = (1 − (ODsample −ODblank)/(ODnegative control − ODblank)) × 100%. The 50% inhibition concentration (IC_50_) for germacrone was determined using GraphPad Prism 6.0 software.

### 2.7. Wound Healing Assay

A wound healing assay was performed as described in the literature [21]. Eca109 and EC9706 cells were seeded in 12-well plates (4 × 105 cells/well) to produce a confluent monolayer. The cell monolayer was scratched with a pipette tip and washed with PBS. Cells were then incubated with different concentrations (0, 5, 10, 15 *μ*g/mL) of germacrone. Moreover, the expression level of matrix metalloproteinases (MMPs) is believed to play an important role in tumor migration and invasion [22]. Scratch wounds were photographed with a phase contrast microscope (Leica, Nussloch, Germany) at 0, 12, and 24 h posttreatment, respectively. The wound area was quantified using ImageJ software.

### 2.8. Flow Cytometry Analysis (FACS)

Eca109 and EC9706 cells were seeded in 6-well plates at a density of 1 × 10^5^ cells/well. After 24 h cultivation, cells were treated with different concentrations (0, 10, 20, 30 *μ*g/mL) of germacrone for 24 h. Cells were then preprocessed and submitted to Annexin V/propidium iodide (PI) staining for FACS analysis according to the protocol described previously [23]. Briefly, cells were harvested by centrifugation (2000 rpm for 5–10 min) and resuspended in 300 *μ*L of 1 × Binding Buffer. Subsequently, 5 *μ*L of Annexin V-FITC and 5 *μ*L of PI solution were added followed by incubation at room temperature for 15 min in the dark. Induction of apoptosis was measured by a Guava easyCyte flow cytometry (Millipore, MA, USA) and analyzed using FlowJo software.

### 2.9. Observation of Morphological Changes

Eca109 and EC9706 cells were seeded in 6-well plates at a density of 1 × 10^5^ cells/well. After 24 h cultivation, cells were treated respectively with germacrone (0, 10, 20, 30 *μ*g/mL) and CCDP (10 *μ*g/mL) for 24 h. The cellular morphological changes following the treatments were observed and photographed under the phase contrast microscope. Cells were then fixed with stationary liquid (methanol: acetic acid = 3 : 1), stained with Hoechst 33258 solution (10 *μ*g/mL), and examined under a fluorescence microscope (Leica, Nussloch, Germany). Cells featured with chromatin condensation, marginalization, or nuclear beading were scored as apoptotic.

### 2.10. Western Blot Analysis

The expression of apoptotic proteins in Eca109 and EC9706 cells after treatment with germacrone was analyzed by Western blot. Briefly, cells were harvested and lysed in RIPA buffer containing the protease inhibitors phenylmethanesulfonyl fluoride and PhosSTOP (Nantong, Jiangsu Province, China). The concentrations for tested proteins in the supernatant obtained by centrifugation (12,000 rpm) of the cell lysate at 4°C were determined by BCA kit (Nantong City, Jiangsu Province, China). The total protein was separated by 7.5–12.5% sodium dodecyl sulfate–polyacrylamide gel electrophoresis (SDS–PAGE) and transferred to a polyvinylidene difluoride membrane (Bio-Rad, CA, USA). The membrane was then blocked with 5% skimmed milk for 2 h at 37°C and probed with primary antibodies overnight at 4°C. After 2 h incubation at 37°C with corresponding secondary antibodies, the protein bands were detected using the HRP-ECL system (Nantong, Jiangsu Province, China).

### 2.11. Statistical Analysis

All data were expressed as means ± standard deviation (SD) of three independent experiments. Statistical analysis was performed with GraphPad Prism 6.0 software using one-way analysis of variance (ANOVA). *P* value <0.05 was considered as statistically significant.

## 3. Results

### 3.1. Germacrone Inhibits ESCC Cell Proliferation

**Figure 1:**
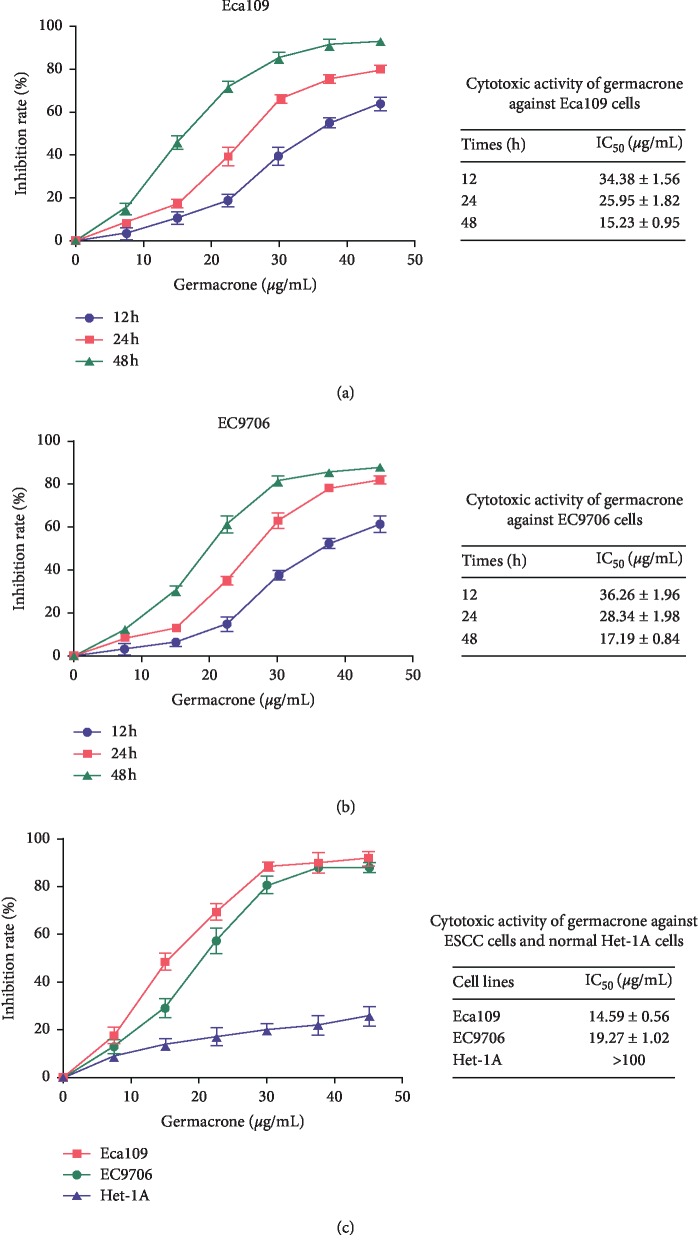
Germacrone inhibits ESCC cell proliferation. (a, b) Eca109 and EC9706 cells were treated with 0, 7.5, 15, 22.5, 30, 37.5, and 45 *μ*g/mL of germacrone for 12, 24, and 48 h (c) Eca109, EC9706, and normal Het-1A cells were treated with 0, 7.5, 15, 22.5, 30, 37.5, and 45 *μ*g/mL of germacrone for 48 h. The inhibition rates and IC_50_ values were determined by MTT assay. Data were presented as means ± SD (*n* = 3).

The effect of germacrone on the viabilities of Eca109 and EC9706 cells was examined by MTT assay. The results revealed that the proliferation of both cell lines were inhibited by germacrone in dose- and time-dependent manners (Figures 1(a) and 1(b)). The IC_50_ values at 12, 24, and 48 h posttreatment were 34.38, 25.95, and 15.23 *μ*g/mL for Eca109 cells and 38.26, 28.34, and 17.19 *μ*g/mL for EC9706 cells, respectively. This suggested that Eca109 cells were slightly more sensitive to germacrone induced growth inhibition than EC9706 cells. Moreover, germacrone exhibited no cytotoxicity against normal Het-1A cells with an IC_50_ higher than 100 *μ*g/mL (Figure 1(c)).

### 3.2. Germacrone Inhibits ESCC Cell Migration

**Figure 2:**
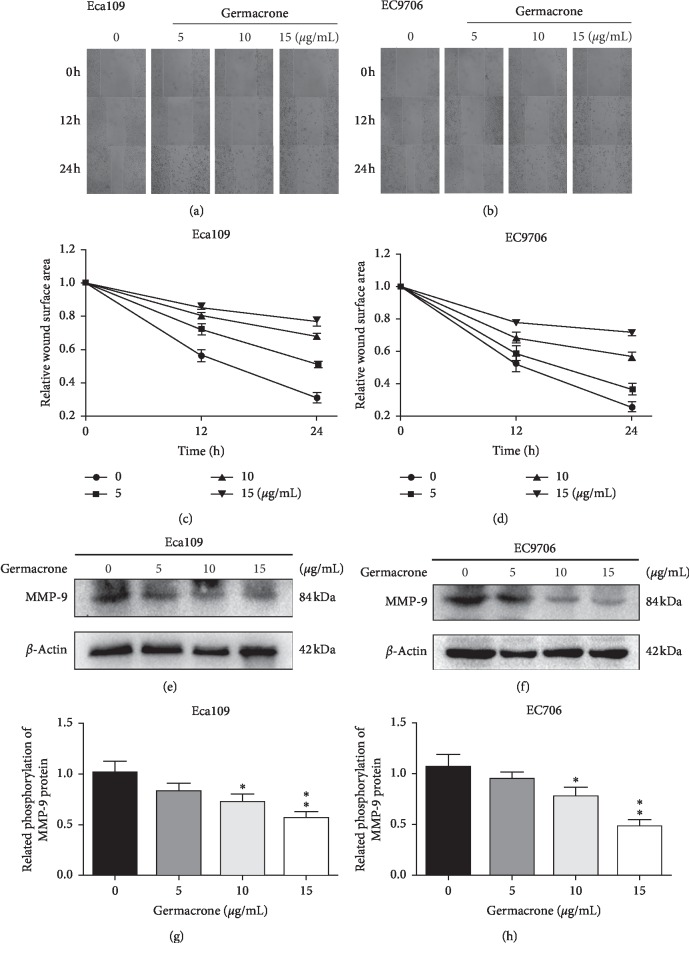
Germacrone inhibits ESCC cell migration. (a, b) Wound healing assays were performed on Eca109 and EC9706 cells. After the creation of the scratch wounds, cells were treated with 0, 5, 10, and 15 *μ*g/mL of germacrone and photographed at 0, 12, and 24 h. (c, d) The relative wound surface area was calculated using ImageJ software. (e)–(h) Western blot analysis of the relative expression of MMP-9 protein in Eca109 and EC9706 cells treated with germacrone for 24 h. Data were presented as means ± SD (*n* = 3). ^*∗*^*P* < 0.05, ^*∗∗*^*P* < 0.01 vs. control (0 *μ*g/mL) group.

The effect of germacrone on the migration capacity of ESCC cells was determined by wound healing experiments. Different concentrations of germacrone and ESCC cells were used to study wound healing. As shown in Figures 2(a) and 2(b), ESCC cells without germacrone treatment were significantly healed after 24 h of wound healing, while ESCC cells treated with germacrone were inhibited, and germacrone had dose-dependent inhibition on wound healing. In addition, after ESCC incubation for 24 h, the relative wound surface area ratio of the germacrone treated group to the blank control group exceeded 0.6. In a molecular mechanism that inhibits cancer cell migration, germacrone dose-dependently inhibits the expression of matrix metalloproteinase (MMP)-9 (Figures 2(e)–2(h)).

### 3.3. Morphological Observation of Apoptosis Induced by Germacrone in ESCC Cells

**Figure 3:**
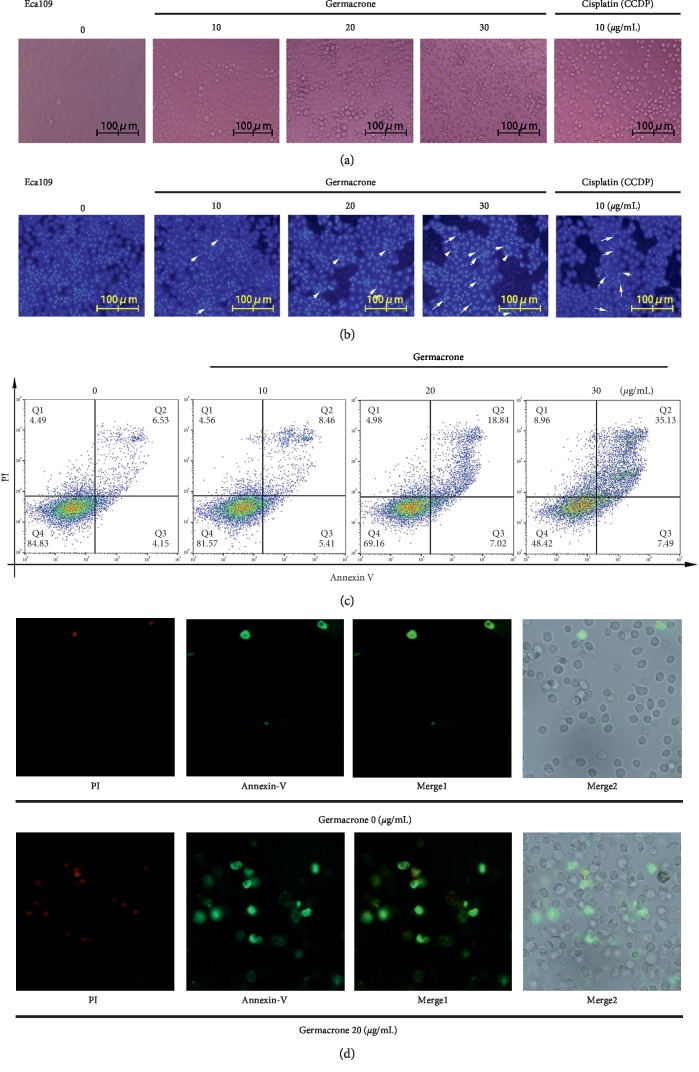
Germacrone induced apoptosis in Eca109 cells. (a) Eca109 cells were treated with germacrone (0, 10, 20, and 30 *μ*g/mL) or CCDP (10 *μ*g/mL) for 24 h. Cellular morphology was observed under a phase contrast microscope. (b) Nuclear morphology was assessed using Hoechst 33258 staining (arrows indicate apoptotic nuclei). (c, d) Apoptosis levels were determined by Flow Cytometry using Annexin V-PI staining.

**Figure 4:**
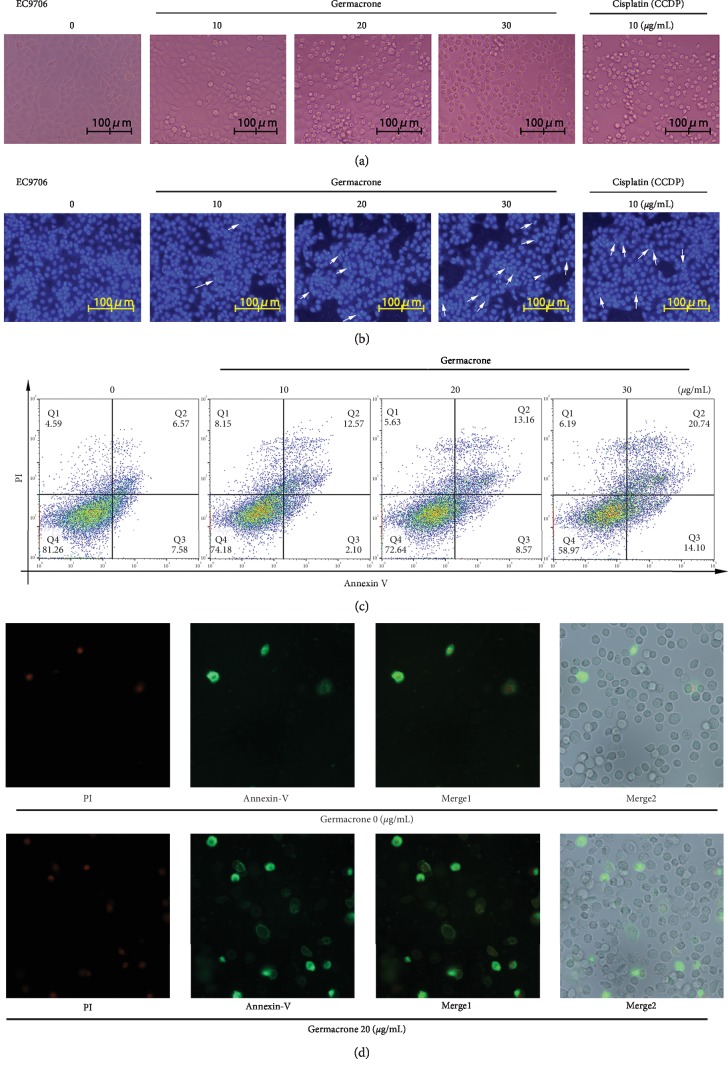
Germacrone induced apoptosis in EC9706 cells. (a) EC9706 cells were treated with germacrone (0, 10, 20, 30 *μ*g/mL) or CCDP (10 *μ*g/mL) for 24 h. Cellular morphology was observed under a phase contrast microscope. (b) Nuclear morphology was assessed using Hoechst 33258 staining (arrows indicate apoptotic nuclei). (c, d) Apoptosis levels were determined by Flow Cytometry using Annexin V-PI staining.

Apoptosis is the most common form of cell death [24]. In order to confirm whether germacrone could induce ESCC cell apoptosis, morphological changes of ESCC cells treated by germacrone or CCDP were observed and compared. When cells undergo apoptosis, some significant morphological changes occur, including fine chromatin condensation and apoptotic bodies [25]. ESCC cells treated with germacrone were observed by phase contrast microscopy. The cell morphology showed typical apoptotic characteristics, such as cell contraction and deformation, and with the increase of germacrone treatment concentration, the number of ESCC cell apoptosis also increased (Figures 3(a)–4(a)). Hoechst 33258 staining was used to observe the accuracy of germacrone for apoptosis of ESCC cells. The results showed that germacrone increased the amount of chromatin condensation and apoptotic bodies in ESCC cells in a dose-dependent manner and was marked with arrows in the photographs (Figures 3(b)–4(b)). To further confirm this result, Annexin V-PI flow cytometry was performed. The results were consistent with the above conclusion that germacrone could significantly induce ESCC (Eca109 and EC9706) cell apoptosis. After incubation with germacrone for 24 h, the apoptosis rates of Eca109 cells were 13.87%, 25.02%, and 42.49%, while the corresponding therapeutic concentrations of germacrone were 10, 20, and 30 *μ*g/mL, respectively (Figure 3(c)). Similarly, in EC9706 cells incubated by germacrone, the apoptosis rates were 17.67%, 21.73%, and 34.84%, respectively (Figure 4(c)). Annexin V-PI staining was used for qualitative analysis. Observation and analysis under the fluorescence microscope revealed that germacrone induced apoptosis in ESCC cells (Figures 3(d)–4(d)). All the above results indicated that germacrone could further inhibit ESCC cell proliferation by inducing ESCC cell apoptosis.

### 3.4. Germacrone Induced ESCC Cell Apoptosis by Intrinsic Apoptosis Pathway

**Figure 5:**
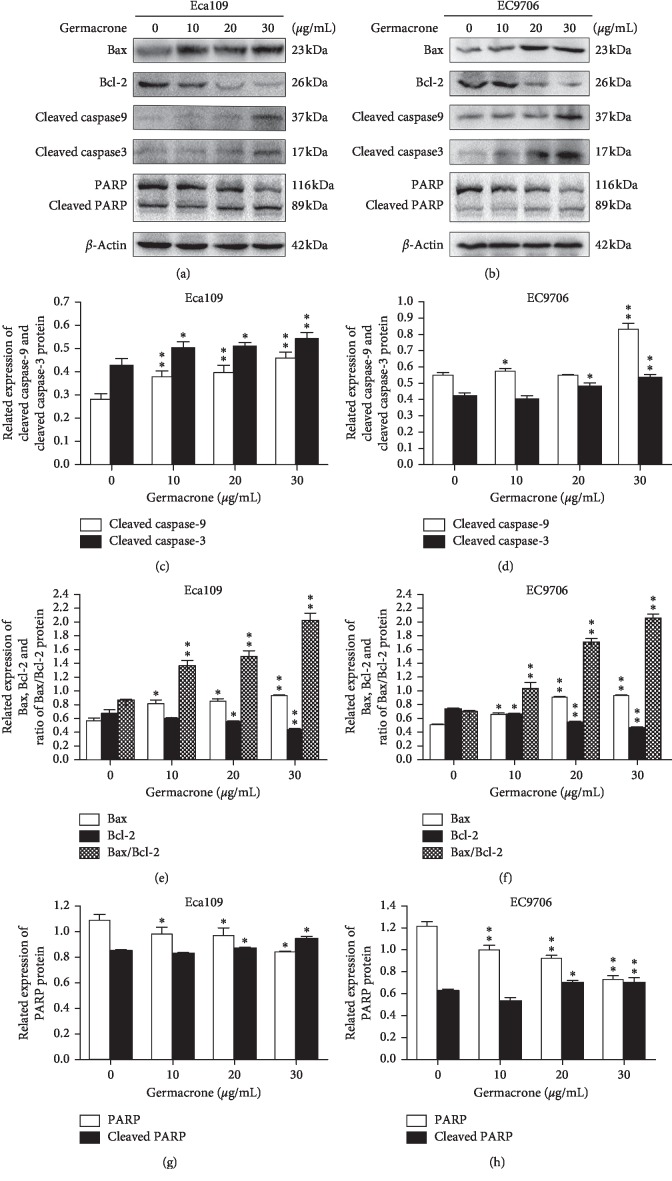
Effect of germacrone on key apoptotic proteins in the mitochondrial apoptotic pathway of ESCC cells. (a)–(h) Western blot analysis of the relative expressions of cleaved Caspase-3, cleaved Caspase-9, PARP, cleaved PARP, Bax and Bcl-2, and the ratio of Bax/Bcl-2 in Eca109 and EC9706 cells treated with germacrone for 24 h. Data were presented as means ± SD (*n* = 3). ^*∗*^*P* < 0.05, ^*∗∗*^*P* < 0.01 vs. control (0 *μ*g/mL) group.

**Figure 6:**
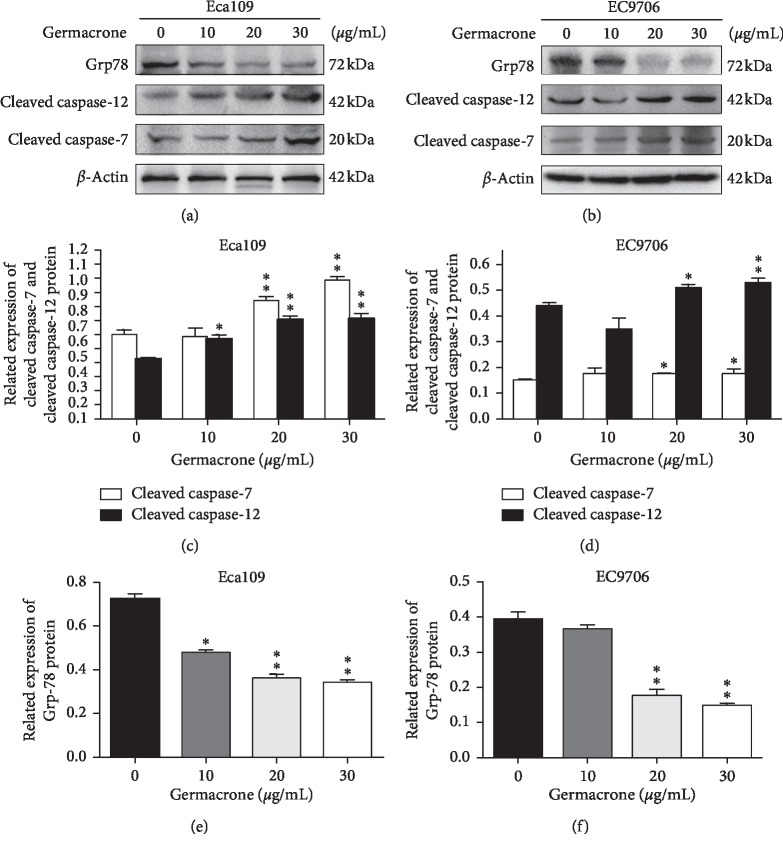
Germacrone inhibits the activity of ERS-related proteins in ESCC cells. (a)–(f) Western blot analysis of the relative expressions of cleaved Caspase-12, cleaved Caspase-7 and Grp78 in Eca109 and EC9706 cells treated with germacrone for 24 h. Data were presented as means ± SD (*n* = 3). ^*∗*^*P* < 0.05, ^*∗∗*^*P* < 0.01 vs. control (0 *μ*g/mL) group.

We are interested in how germacrone affects the expression of key proteins in the apoptosis mechanism. Proapoptotic Bax and antiapoptotic Bcl-2 regulate intrinsic apoptosis senses of death signals [26]. Previous studies have reported that the balance of Bax/Bcl-2 determines the occurrence of mitochondrial apoptosis and the increase of Bax/Bcl-2 is considered to be the starting site for promoting apoptosis [27]. Caspase-9 and Caspase-3 are critical for mitochondrial apoptosis in cells, and once they are activated, their precursor forms are cleaved [28]. PARP is the target protein of Caspase-3, affecting the repair of cancer cells. Activated Caspase-3 cleaves PARP and prevents DNA from repair [29]. Therefore, we used western blot to detect the expression of five key proteins (Bcl-2, Bax, cleaved Caspase-9, cleaved PARP, and cleaved Caspase-3) in ESCC cells after incubation with germacrone. And germacrone increased the proportion of Bax/Bcl-2 and the expression of cleaved Caspase-9, Caspase-3, and PARP in ESCC cells in a dose-dependent manner (Figure 5). These results suggest that germacrone may induce apoptosis in ESCC cells by activating the mitochondrial apoptotic pathway. In addition, Caspase-3 may be activated through the endoplasmic reticulum apoptosis pathway, so we also studied the effect of germacrone on the endoplasmic reticulum of ESCC cells. Persistence of endoplasmic reticulum stress (ERS) induces apoptosis [30]. Caspase-12 plays an indispensable role in ERS-induced apoptosis, and Caspase-7 mediates apoptosis through translocation and cleavage [31]. Nevertheless, inhibition of ERS-induced apoptosis is usually prevented by the glucose regulatory protein Grp78 from the activation of Caspase [32]. Therefore, we investigated the expression of Grp78, cleaved Caspase-12, and cleaved Caspase-7 in germacrone treated ESSC cells. The results showed that germacrone could increase the expression of Caspase-12 and Caspase-7 lysed in ESSC cells and downregulate the expression of Grp78 (Figure 6). The experimental results indicate that germinone-induced apoptosis of ESCC cells is also mediated by stimulation of the endoplasmic reticulum signaling pathway. Therefore, combining all of the above findings, we conclude that germacrone induces apoptosis in ESCC cells via the intrinsic apoptotic signaling pathway.

### 3.5. Germacrone Represses STAT3 Activity in ESCC Cells

**Figure 7:**
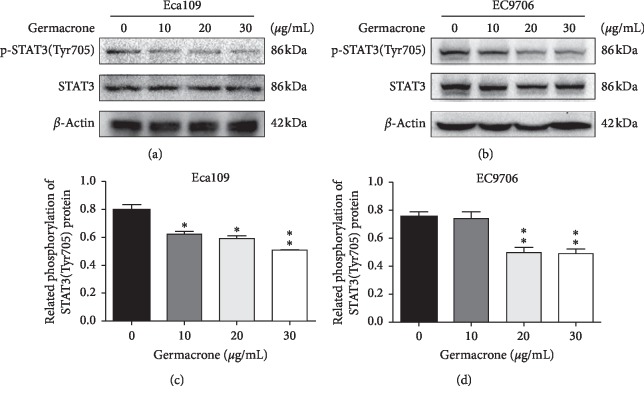
Germacrone repressed STAT3 activity in ESCC cells. (a)–(d) Western blot analysis of STAT3 phosphorylation on Tyr705 in Eca109 and EC9706 cells treated with germacrone for 24 h. Data were presented as means ± SD (*n* = 3). ^*∗*^*P* < 0.05, ^*∗∗*^*P* < 0.01 vs. control (0 *μ*g/mL) group.

STAT3 is one of the most important transcription factors in the growth of cells, regulating cell proliferation and apoptosis, and is continuously activated in human cancer cells [33]. In addition, Bcl-2 is one of the sites of action of the STAT3 gene [34]. In view of the fact that germacrone has been shown to downregulate Bcl-2 protein in ESCC cells in the above experiments, we also have an interest in germacrone in the changes in STAT3 protein expression in ESCC cells. Previous studies have found that phosphorylated STAT3 active forms are primarily mediated by phosphorylation at Tyr705 [35]. Hence, the expressions of STAT3 were investigated (Figures 7(a) and 7(b)). As shown in the results of Figures 7(c) and 7(d), germacrone dose-dependently inhibits basal phosphorylation of STAT3 at Tyr705 but does not affect the protein level of total STAT3. Therefore, these findings indicate that germacrone has the potential to inhibit STAT3 phosphorylation in ESCC cells.

## 4. Discussion

Some studies have shown that the anticancer activity of *Saussurea costus* is mainly due to its major components sesquiterpenoids [36, 37]. Germacrone is a sesquiterpene and has certain anticancer effects on glioma, retinoblastoma, breast cancer, liver cancer, prostate cancer, and colon cancer [12–17, 38]. However, the pharmacological effects and mechanisms of germacrone against human ESCC are still poorly understood. In our study, germacrone showed antiproliferative activity against two ESCC cell lines. Our research manifested that germacrone dose-dependently and time-dependently inhibited ESCC cell proliferation and migration. Therefore, we conducted in *vitro* studies to further elucidate the antitumor potential of germacrone in ESCC cells.

The typical pathological hallmarks of ESCC cells, like most malignant tumor cells, are invasion, metastasis, and resistance to apoptosis [39–41]. Our study showed that germacrone treatment inhibited the migration of Eca109 and EC9706 cells even at a low concentration and inhibited the expression of matrix metalloproteinase dose-dependently, suggesting that germacrone can inhibit the invasion and migration of ESCC cells. Apoptosis is an intrinsic self-regulatory mechanism associated with many biological processes. Defects in apoptosis mechanisms can destroy the delicate balance of the cell process and might eventually lead to cancer. Some cancers, including ESCC, are highly dependent on survival abnormalities in the apoptotic signaling pathway, but still retain some of the apoptotic mechanisms. Therefore, targeted therapy for the development of natural drugs by inducing apoptosis in cancer cells is crucial. Therefore, we first determined whether germacrone would induce apoptosis in ESCC cells through morphological observation.

The apoptotic pathways can be divided into intrinsic apoptotic pathways and extrinsic apoptotic pathways. Mitochondria play an important role in intrinsic apoptotic pathways [42]. Bcl-2 family members are very pivotal in regulating this pathway. The relative expression of Bcl-2 and Bax and the activation of Caspase-9 and Caspase-3 are classical events mediating mitochondrial apoptosis signaling pathway, among which Bax and Bcl-2 regulate the apoptotic activators by controlling the permeability of mitochondrial membrane [43]. Increased Bax/Bcl-2 ratio in cells leads to the release of mitochondrial pigment C, which activates caspase-9 and caspase-3, and ultimately induces cell apoptosis. PARP is an early target of activated Caspase, and its cleavage product can be used as a marker of apoptosis [44]. FACS analysis showed that the proportion of apoptotic cells increased gradually after germacrone treatment, which was consistent with the observation of changes in apoptotic bodies under phase contrast microscopy. Additionally, western blot analysis showed that germacrone treatment dose-dependently decreased cleaved PARP levels, suggesting that germacrone induced apoptosis in ESCC cells, which was verified by other western blot results. The ratio of Bax/Bcl-2 was dose-dependently increased after germacrone treatment. These results illustrated that germacrone may be a potential anti-ESCC agent by activating the mitochondrial apoptotic signaling pathway. Secondly, endoplasmic reticulum also plays a role in the intrinsic apoptosis pathway. Since the upstream sites of Caspase-3 activation and Caspase-12 activation were also involved, we were curious about whether germacrone would affect the endoplasmic reticulum to induce apoptosis. And we found that the endoplasmic reticulum apoptosis pathway is a newly discovered apoptosis pathway. ERS is triggered by an imbalance in calcium homeostasis and accumulation of unfolded or misfolded proteins in the ER. The persistence of ERS induces normal cell apoptosis, but in the early ERS, the cells evoke a repair mechanism called the unfolded protein response (UPR) to reconstruct ER homeostasis in an attempt to maintain viability. When cancer cells are exposed to metabolic disorders such as hypoxia, nutrient deficiency, and oxidative stress, this leads to persistent ERS. However, cancer cells appear to benefit from UPR to escape apoptosis and adapt to the transformed state [45]. Considering that the upregulation of ERS is also detected in ESCC and is associated with the development of ESCC [46], we examined the impact of germacrone on ERS in ESCC. Grp78 expression was detected in germacrone treated Eca109 and EC9706 cells, and the increased expression of Grp78 is usually associated with the initiation of microenvironmental stress and UPR in a variety of cancers. The dose-dependent inhibition of germacrone on Grp78 expression indicated that it had an effect on the ERS pathway, which stimulated our interest in the involvement of the ERS pathway in germacrone induced apoptosis of ESCC cells. Caspase-12, as a core molecule, is activated by Grp78 and Caspase-7, which is related to the ERS apoptosis signaling pathway [31]. The expressions of cleaved caspase-12 and cleaved caspase-7 were detected, and the results showed that germacrone treatment increased their activity. These findings suggested that germacrone induces apoptosis by inducing the intrinsic apoptosis pathway of ESCC cells.

In general, the mechanism of apoptosis is also controlled by some antiapoptotic genes. The previous studies on the antiproliferation and migration of ESCC cells by germacrone have motivated us to imagine whether germacrone affects cell proliferation, migration, and apoptosis by controlling certain genes in ESCC. Germacrone inhibits cell proliferation by affecting the division process of ESCC cells and it inhibits cell migration and invasion by inhibiting the expression of MMP family in ESCC cells. In studying the intrinsic apoptosis pathway, we evaluated the effect of germacrone on the Bcl-2 family of ESCC cells. Based on the above studies, we hypothesized that germacrone is sensitive to the STAT3 gene in ESCC. STAT3 is a key molecule in the antiapoptotic pathway important in many cancers [47]. To investigate whether STAT3 signaling was a germacrone-triggered ESCC apoptotic medium, STAT3 activity was tested *in vitro*. The result showed that germacrone had a dose-dependent effect on STAT3 activity. This result improves our understanding of the importance of germacrone in the treatment of ESCC cells and suggests that germacrone may be a STAT3 inhibitor in the development of targeted chemotherapy drugs.

## 5. Conclusions

In conclusion, germacrone, a natural 10-membered monocyclic sesquiterpene with three double bonds and a ketone, was isolated and identified from the roots of *Saussurea costus*. Germacrone has the capacity of inducing ESCC cell apoptosis via affecting Caspase-dependent mitochondria, ER stress, and STAT3 inactivation. This natural product may have the potential to be developed into a new therapeutic drug against ESCC.

## Data Availability

The data used to support the findings of this study are available from the corresponding author upon request.

## Supplementary Materials

Supplementary Materials The 1HNMR and 13CNMR spectra of Germacrone. S1: the chemical structure and purity of germacrone. S2: 1HNMR spectrum (600 MHz, CDCl3) of germacrone. S3: 13CNMR spectrum (150 MHz, CDCl3) of germacrone. S4: NMR spectroscopic data of germacrone.

## References

[1] Bray F., Ferlay J., Soerjomataram I., Siegel R. L., Torre L. A., Jemal A. (2018). Global cancer statistics 2018: globocan estimates of incidence and mortality worldwide for 36 cancers in 185 countries. *CA: A Cancer Journal for Clinicians*.

[2] Pennathur A., Gibson M. K., Jobe B. A., Luketich J. D. (2013). Oesophageal carcinoma. *The Lancet*.

[3] Ferlay J., Steliarova-Foucher E., Lortet-Tieulent J. (2013). Cancer incidence and mortality patterns in Europe: estimates for 40 countries in 2012. *European Journal of Cancer*.

[4] Mcguire S. (2016). World cancer report 2014. Geneva, Switzerland: world Health organization, international agency for research on cancer, who press, 2015. *Advances in Nutrition*.

[5] Colquhoun A., Arnold M., Ferlay J., Goodman K. J., Forman D., Soerjomataram I. (2015). Global patterns of cardia and non-cardia gastric cancer incidence in 2012. *Gut*.

[6] Gordaliza M. (2007). Natural products as leads to anticancer drugs. *Clinical and Translational Oncology*.

[7] Mann J. (2002). Natural products in cancer chemotherapy: past, present and future. *Nature Reviews Cancer*.

[8] Pandey M. M., Rastogi S., Rawat A. K. S. (2007). Saussurea Costus: botanical, chemical and pharmacological review of an ayurvedic medicinal plant. *Journal of Ethnopharmacology*.

[9] Tabata K., Nishimura Y., Takeda T., Kurita M., Uchiyama T., Suzuki T. (2015). Sesquiterpene lactones derived from Saussurea lappa induce apoptosis and inhibit invasion and migration in neuroblastoma cells. *Journal of Pharmacological Sciences*.

[10] Chen H. C., Chou C. K., Lee S. D., Wang J. C., Yeh S. F. (1995). Active compounds from Saussurea lappa clarks that suppress hepatitis B virus surface antigen gene expression in human hepatoma cells. *Antiviral Research*.

[11] Chhabra B. R., Gupta S., Jain M., Kalsi P. S. (1998). Sesquiterpene lactones from Saussurea lappa. *Phytochemistry (Oxford)*.

[12] Liu B., Gao Y.-Q., Wang X.-M., Wang Y.-C., Fu L.-Q. (2014). Germacrone inhibits the proliferation of glioma cells by promoting apoptosis and inducing cell cycle arrest. *Molecular Medicine Reports*.

[13] Chokchaisiri R., Pimkaew P., Piyachaturawat P., Chalermglin R., Suksamrarn A. (2014). Cytotoxic sesquiterpenoids and diarylheptanoids from the rhizomes of curcuma elata roxb. *Records of Natural Products*.

[14] Xie X.-H., Zhao H., Hu Y.-Y., Gu X.-D. (2014). Germacrone reverses Adriamycin resistance through cell apoptosis in multidrug-resistant breast cancer cells. *Experimental and Therapeutic Medicine*.

[15] Zhong Z. F., Chen X. P., Tan W. (2011). Germacrone inhibits the proliferation of breast cancer cell lines by inducing cell cycle arrest and promoting apoptosis. *European Journal of Pharmacology*.

[16] Ahmed Hamdi O. A., Syed Abdul Rahman S. N., Awang K. (2014). Cytotoxic constituents from the rhizomes of Curcuma zedoaria. *The Scientific World Journal*.

[17] Liu Y. Y., Wang W., Fang B. (2013). Anti-tumor effect of germacrone on human hepatoma cell lines through inducing G2/M cell cycle arrest and promoting apoptosis. *European Journal of Pharmacology*.

[18] Lee S. O., Choi S. Z., Choi S. U., Kim G. H., Kim Y. C., Lee K. R. (2006). Cytotoxic terpene hydroperoxides from the aerial parts ofAster spathulifolius. *Archives of Pharmacal Research*.

[19] Kumar P., Nagarajan A. (2018). Analysis of cell viability by the mtt assay. *Cold Spring Harbor Protocols*.

[20] Yang J., Chen H., Wang Q., Deng S. H., Huang M. .. (2018). Inhibitory effect of kurarinone on growth of human non-small cell lung cancer: an experimental study both in vitro and in vivo studies. *Frontiers in Pharmacology*.

[21] Liang C.-C., Park A. Y., Guan J.-L. (2007). In vitro scratch assay: a convenient and inexpensive method for analysis of cell migration in vitro. *Nature Protocols*.

[22] Jiang B., Chen J., Yuan W. (2018). Platelet-derived growth factor-D promotes colorectal cancer cell migration, invasion and proliferation by regulating Notch1 and matrix metalloproteinase-9. *Oncology Letters*.

[23] Khazaei S., Esa N. M., Ramachandran V. (2017). In vitro antiproliferative and apoptosis inducing effect of allium atroviolaceum bulb extract on breast, cervical, and liver cancer cells. *Frontiers in Pharmacology*.

[24] Hetz C. (2008). Editorial (apoptosis, necrosis and autophagy: from mechanisms to biomedical applications (part-I) guest editor: claudio hetz). *Current Molecular Medicine*.

[25] Ihara T., Yamamoto T., Sugamata M., Okumura H., Ueno Y. (1998). The process of ultrastructural changes from nuclei to apoptotic body. *Virchows Archiv*.

[26] Fox J. L., MacFarlane M. (2016). Targeting cell death signalling in cancer: minimising “Collateral damage”. *British Journal of Cancer*.

[27] Ghate N. B., D Chaudhuri A. D.., Panja S., Mandal N. (2016). Sundew plant, a potential source of anti-inflammatory agents, selectively induces G2/M arrest and apoptosis in mcf-7 cells through upregulation of P53 and bax/bcl-2 ratio. *Cell Death Discovery*.

[28] Lopez J., Tait S. W. G. (2015). Mitochondrial apoptosis: killing cancer using the enemy within. *British Journal of Cancer*.

[29] Henning R. J., Bourgeois M., Harbison R. D. (2018). Poly(Adp-Ribose) polymerase (parp) and parp inhibitors: mechanisms of action and role in cardiovascular disorders. *Cardiovascular Toxicology*.

[30] Szegezdi E., Logue S. E., Gorman A. M., Samali A. (2006). Mediators of endoplasmic reticulum stress—induced apoptosis. *EMBO Reports*.

[31] Nakagawa T., Zhu H., Morishima N. (2000). Caspase-12 mediates endoplasmic-reticulum-specific apoptosis and cytotoxicity by amyloid-*β*. *Nature*.

[32] Rao R. V., Peel A., Logvinova A. (2002). Coupling endoplasmic reticulum stress to the cell death program: role of the Er chaperone Grp78. *FEBS Lett*.

[33] Yu H., Jove R. (2004). The STATs of cancer - new molecular targets come of age. *Nature Reviews Cancer*.

[34] Avalle L., Pensa S., Regis G., Novelli F., Poli V. (2012). STAT1 and STAT3 in tumorigenesis. *JAK-STAT*.

[35] Jia Z.-H., Jia Y., Guo F.-J., Chen J., Zhang X.-W., Cui M.-H. (2017). Phosphorylation of Stat3 at Tyr705 regulates MMp-9 production in epithelial ovarian cancer. *PLoS One*.

[36] Sieniawska E., Świątek Ł., Rajtar B., Kozioł E., Polz-Dacewicz M., Skalicka-Woźniak K. (2016). Carrot seed essential oil-Source of carotol and cytotoxicity study. *Industrial Crops and Products*.

[37] Pudziuvelyte L., Stankevicius M., Maruska A. (2017). Chemical composition and anticancer activity of elsholtzia ciliata essential oils and extracts prepared by different methods. *Industrial Crops and Products*.

[38] Kong Q., Ma Y., Yu J. (2017). Predicted molecular targets and pathways for germacrone, curdione, and furanodiene in the treatment of breast cancer using a bioinformatics approach. *Scientific Reports*.

[39] Hara F., Aoe M., Doihara H. (2005). Antitumor effect of gefitinib (“Iressa”) on esophageal squamous cell carcinoma cell lines in vitro and in vivo. *Cancer Letters*.

[40] Sun S.-J., Feng L., Zhao G.-Q., Dong Z.-M. (2012). Retracted article: HAX-1 promotes the chemoresistance, invasion, and tumorigenicity of esophageal squamous carcinoma cells. *Digestive Diseases and Sciences*.

[41] Wang Q., Pan L.-H., Lin L. (2018). Essential oil from carpesium abrotanoides L. Induces apoptosis via activating mitochondrial pathway in hepatocellular carcinoma cells. *Current Medical Science*.

[42] Yin X.-M. (2000). Signal transduction mediated by bid, a pro-death bcl-2 family proteins, connects the death receptor and mitochondria apoptosis pathways. *Cell Research*.

[43] Chen H., Zhou B., Yang J. (2018). Essential oil derived from eupatorium adenophorum spreng. Mediates anticancer effect by inhibiting Stat3 and akt activation to induce apoptosis in hepatocellular carcinoma. *Frontiers in Pharmacology*.

[44] Yokoyama T., Kohn E. C., Brill E., Lee J. M. (2017). Apoptosis is augmented in high-grade serous ovarian cancer by the combined inhibition of Bcl-2/Bcl-xL and PARP. *International Journal of Oncology*.

[45] Wang G. H., Yang Z. Q., Zhang K. Z. (2010). Endoplasmic reticulum stress response in cancer: molecular mechanism and therapeutic potential. *American Journal of Translational Research*.

[46] Fan C., Yang Y., Liu Y., Jiang S., Di S. (2016). Icariin displays anticancer activity against human esophageal cancer cells via regulating endoplasmic reticulum stress-mediated apoptotic signaling. *Scientific Reports*.

[47] Yu H., Pardoll D., Jove R. (2009). Stats in cancer inflammation and immunity: a leading role for Stat3. *Nature Reviews Cancer*.

