# Supplementary material for: Germacrone Inhibits Cell Proliferation and Induces Apoptosis in Human Esophageal Squamous Cell Carcinoma Cells
Source: Biomed Res Int. 2020 Jan 30;2020:7643248. doi: 10.1155/2020/7643248 (PMC7011320; doi:10.1155/2020/7643248)
Supplement: Supplementary Materials — The 1HNMR and 13CNMR spectra of Germacrone. S1: the chemical structure and purity of germacrone. S2: 1HNMR spectrum (600 MHz, CDCl3) of germacrone. S3: 13CNMR spectrum (150 MHz, CDCl3) of germacrone. S4: NMR spectroscopic data of germacrone. [file 7643248.f1.doc]

Germacrone Inhibits Cell Proliferation and Induces Apoptosis in Human Esophageal Squamous Cell Carcinoma Cells

**Ren Zhang^1^, Ji Hao^2^, Kaiwen Guo^1^, Wanxin Liu^1^, Fei Yao^1^, Qingming Wu^1^, Chang Liu^2^, Qiang Wang^1*^ and Xinzhou Yang^2*^**

*^1^ Institute of Infection, Immunology and Tumor Microenviroment, Hubei Province Key Laboratory of Occupational Hazard Identification and Control, Medical College, Wuhan University of Science and Technology,* *Wuhan 430065, China*

*^2^ School of Pharmaceutical Sciences, South-Central University for Nationalities, Wuhan 430074, China*

*Correspondence should be addressed to Qiang Wang; wangqiang@wust.edu.cn and Xinzhou Yang; xzyang@mail.scuec.edu.cn

**List of contents**

**S1. The chemical structure and purity of germacrone**

**S2.** **^1^H NMR spectrum (600 MHz, CDCl_3_) of germacrone**

**S3. ^13^C NMR spectrum (150 MHz, CDCl_3_) of germacrone**

**S4. NMR spectroscopic data of germacrone**

**
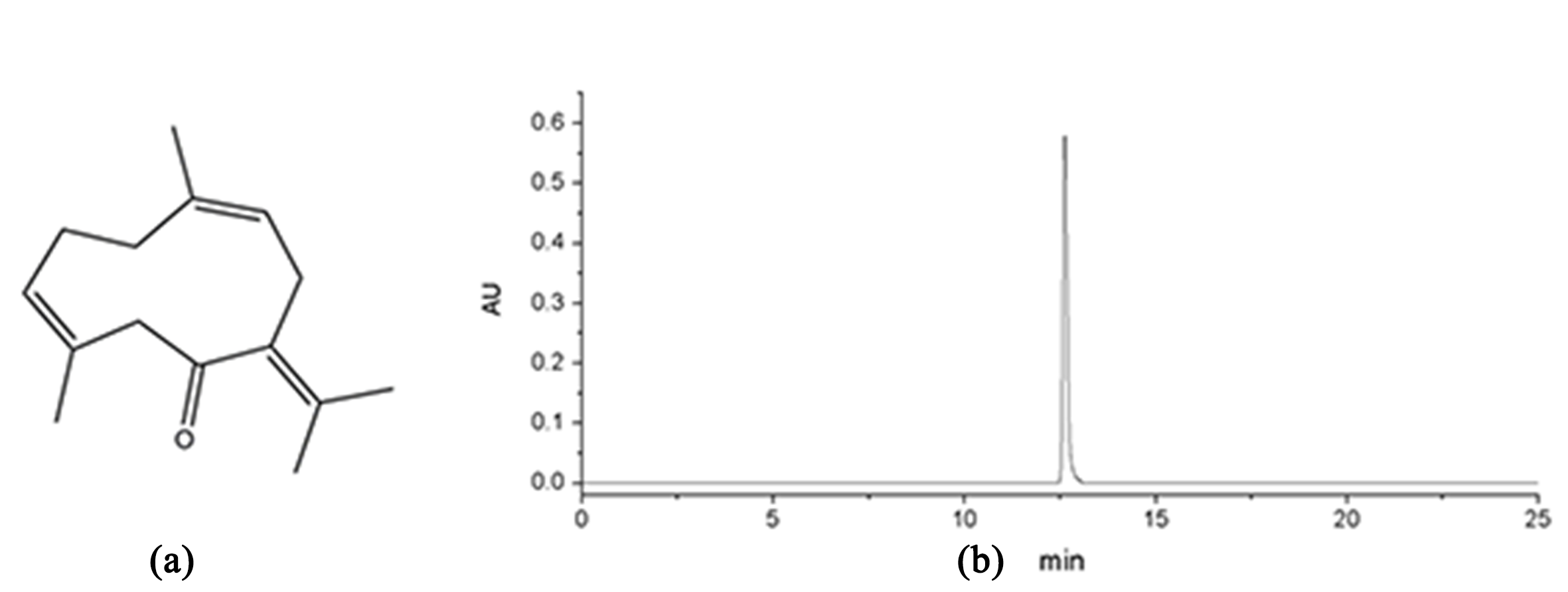
** **Figure S1. (a) Chemical structure of germacrone; (b) HPLC chromatogram of germacrone.**


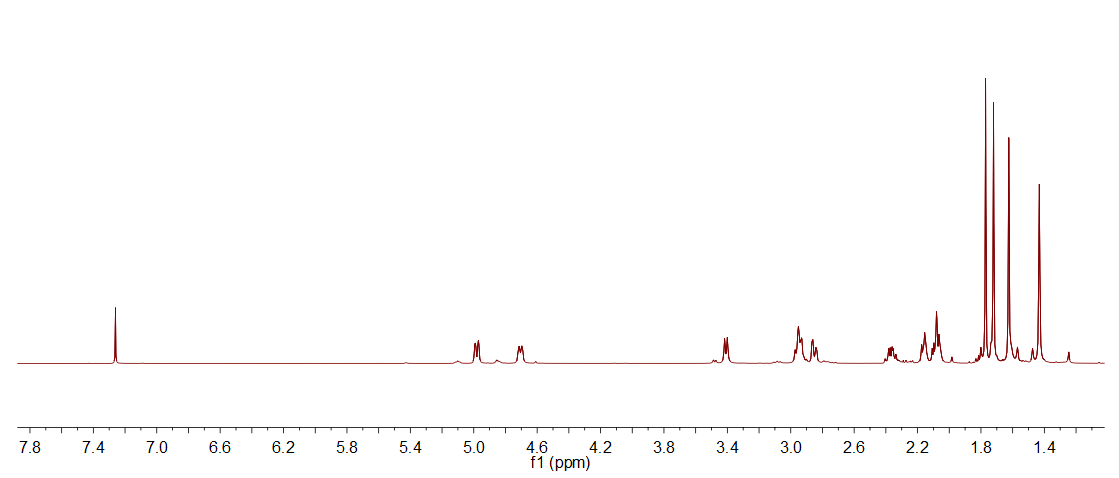


**Figure S2. ^1^H NMR spectrum of germacrone (600 MHz, CDCl_3_)**


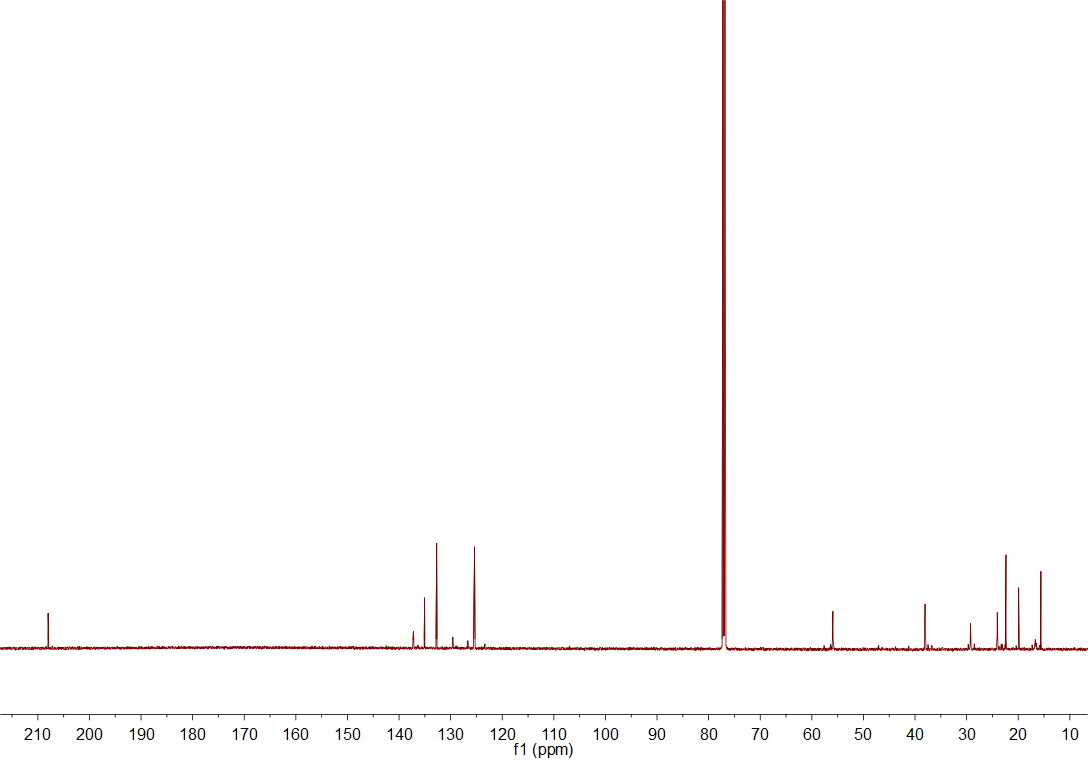


**Figure S3. ^13^C NMR spectrum of germacrone (150 MHz, CDCl_3_)**

**S4. NMR spectroscopic data of germacrone**

Germacrone: colorless needles; ^1^H NMR (CDCl_3_, 600 MHz): δ_H_ 4.98 (1H, d, *J* = 11.5 Hz, H-1), 4.71 (1H, d, *J* = 9.7 Hz, H-5), 3.41 (1H, d, *J* =10.4 Hz, H-9α), 2.95 (2H, m, H-6α, 9β), 2.85 (1H, d, *J* = 13.5Hz, H-6β), 2.05～2.40 (4H, m, H-2, 3), 1.77 (3H, s, H-13), 1.72 (3H, s, H-12), 1.63 (3H, s, H-15), 1.43 (3H, s, H-14); ^13^C NMR (CDCl_3_, 150 MHz): δ_C_ 208.1 (C-8), 137.3 (C-11), 135.2 (C-10), 132.8 (C-1), 129.7 (C-7), 126.8 (C-4), 125.5 (C-5), 56.1 (C-9), 38.2 (C-3), 29.4 (C-6), 24.2 (C-2), 22.5 (C-13), 20.1 (C-12), 16.7 (C-15), 15.7 (C-14). ESIMS: 219 [M+H]^+^, 217 [M-H]^-^. The spectroscopic data were identical with those reported in the literature [1].

[1] S.O. Lee, S.Z. Choi et al.,“Cytotoxic Terpene Hydroperoxides from the Aerial Parts of Aster Spathulifolius,” *Arch Pharm Res*, vol. 29, no. 10, pp. 845-8, 2006.
